# Strangulated Inguinal Hernia in Infants with Report of a Case

**Published:** 1907-11

**Authors:** John Egerton Cannaday

**Affiliations:** Hansford, W. Va„ Surgeon-in-Charge, Sheltering Arms Hospital


### Strangulated Inguinal Hernia in Infants with Report of a Case.

BY JOHN EGERTON CANNADAY, M. D., Hansford, W. Va.,
Surgeon-in-Charge, Sheltering Arms Hospital.

*(Author's Abstract)*

THE author discusses the subject of inguinal hernia in infants and concludes that it is best to advise the wearing of a truss for several years of infancy and early childhood, with the hope of getting a cure without an operation—only resorting to operation in case of strangulation or of failure to effect a cure by the use of a truss.

When strangulation does occur, operative relief is even more imperative than in the case of an adult, as the powers of endurance are less. The pathology and symptomatology, diagnosis, prognosis, and treatment of strangulated hernia is considered. The author does not believe in wasting much time in taxis. He gives indication for reduction or resection of intestines. He objects to transplantation of the cord by reason of its predisposition to epididymitis. He advises the use of buried sutures of 30-day chromic catgut. The skin edges are brought together by a subcutaneous suture of celluloid linen thread.

The author reports a case of strangulated hernia in a three-months old male baby. Reduction was attempted under chloroform anesthesia before patient was brought to the hospital. The operation was performed under local anesthesia induced by a 2 per cent. solution of eucaine; a collodion dressing was used. Cannaday summarizes as follows:

I would note the infrequent but occasional occurrence of strangulated hernia in infants. The ease of a reasonable certainty in the diagnosis of this condition. The dangers of operation in infants have been overestimated. The dangers of taxis by reason of the fragility of the structures involved. By proper precaution and care the risks of post-operative infection can be largely eliminated. The shock incident to anesthesia and prolonged manipulation can be avoided by the proper use of a sterile and practically nontoxic local anesthetic and by rapid and careful work.

